# Culturally Adapting an Internet-Delivered Mindfulness Intervention for Indonesian University Students Experiencing Psychological Distress: Mixed Methods Study

**DOI:** 10.2196/47126

**Published:** 2023-08-31

**Authors:** Ratih Arruum Listiyandini, Annisa Andriani, Chandradewi Kusristanti, Michelle Moulds, Alison Mahoney, Jill M Newby

**Affiliations:** 1 School of Psychology Faculty of Science University of New South Wales Sydney Australia; 2 Faculty of Psychology Universitas YARSI Jakarta Pusat, DKI Jakarta Indonesia; 3 Faculty of Psychology Universitas Jambi Jambi Indonesia; 4 Clinical Research Unit for Anxiety and Depression St Vincent's Hospital Sydney Australia; 5 School of Psychiatry University of New South Wales Sydney Australia; 6 Black Dog Institute Sydney Australia

**Keywords:** psychological distress, mindfulness, cultural adaptation, internet-delivered, students, Indonesia, mobile phone

## Abstract

**Background:**

Psychological distress is prevalent among university students. However, the availability of evidence-based mental health treatment remains limited in many low- and middle-income countries, including Indonesia. Internet-delivered, mindfulness-based interventions that reduce distress have potential for treating university student distress at scale. Unfortunately, evidence-based, internet-delivered mindfulness treatments are not yet available in Indonesia. Cultural adaptation of established evidence-based, internet-delivered mindfulness interventions is needed.

**Objective:**

In this paper, we describe the process of culturally adapting an Australian internet-delivered mindfulness program (*Introduction to Mindfulness*) to be relevant and appropriate for treating Indonesian university students’ psychological distress.

**Methods:**

To assist the cultural adaptation process, we used a systematic cultural adaptation framework and a mixed methods approach combining quantitative and qualitative methods. In study 1 (information gathering), we administered an internet-delivered questionnaire to Indonesian university students (n=248) to examine their preferences regarding an internet-delivered mindfulness intervention. In study 2 (preliminary design), a draft program was developed and independently reviewed by Indonesian stakeholders. Stakeholders (n=25) included local Indonesian mindfulness and mental health professionals (n=6) and university students (n=19), who were selected to maximize sample representativeness regarding personal and professional characteristics. To evaluate the initial design and cultural congruence of the internet-delivered mindfulness program in the Indonesian context, we conducted interviews and focus groups with stakeholders. Stakeholders also completed the Cultural Relevance Questionnaire.

**Results:**

In study 1, most Indonesian university students (240/248, 96.8%) reported openness to an internet-delivered mindfulness program. Most of interested students (127/240, 52.9%) preferred the length of the program to be 3 to 4 sessions, with 45.8% (110/240) preferring brief lessons taking only 15 to 30 minutes to complete. They (194/240, 80.8%) recommended that the program be accessible both through websites and mobile phones. In study 2, Indonesian stakeholders generally found the internet-delivered program to be highly culturally appropriate in terms of language, concepts, context, treatment goals, and depictions of students’ emotional and behavioral experiences. However, stakeholders also recommended some specific adaptations regarding the program’s delivery model (eg, combining visual and audio modalities when delivering psychoeducation), cultural components (eg, including more social and spiritual activities), program practicality (eg, including rewards to promote engagement), and design elements (eg, including additional culturally relevant illustrations). Following stakeholder feedback, a new culturally adapted Indonesian internet-delivered mindfulness program called *Program Intervensi Mindfulness Daring Mahasiswa Indonesia* was created.

**Conclusions:**

This study highlights the process and importance of cultural adaptation of an evidence-based mindfulness treatment and demonstrates how this may be achieved for internet-delivered psychotherapy programs. We found that a culturally adapted internet-delivered mindfulness program was relevant for Indonesian students with some adjustments to the programs’ content and delivery. Future research is now needed to evaluate the clinical benefit of this program.

## Introduction

### Background

The World Mental Health International College Student survey assessed mental health in >13,000 full-time university students and found that approximately 30% of respondents screened positive for at least one mental health problem, such as depression or anxiety [1,2]. Unfortunately, access to evidence-based psychological treatment in low- and middle-income countries (LMICs) such as Indonesia is limited. In Indonesia, there are few trained mental health professionals, most of whom are in urban rather than rural areas [3]. The ratio of registered clinical psychologists in Indonesia is only 1.3 per 100,000 people (3602 registered clinical psychologists for 260 million people), with 80% of them working on the Java island [4]. Negative cultural beliefs about emotional problems [5] and stigma toward mental illness [6] also still exist in Indonesia.

Delivering mental health interventions using the internet may offer several advantages [7,8]. Internet-based delivery may reduce treatment barriers related to cost, transportation, availability of service, wait time, and stigma and, thus, help reduce health care–related disparities [9,10]. It is also in line with the development of digital penetration in Indonesia, which is said to reach >70% according to the Indonesian Internet Service Provider Association [11]. However, to date, only 1 randomized controlled trial using behavioral activation for depression [12] and 2 feasibility studies of cognitive and behavioral therapies for university students [12-14] have been conducted among Indonesian populations. Although these studies support the utility of internet-delivered mental health interventions in Indonesian populations, further studies are needed to explore other culturally appropriate therapy approaches.

Mindfulness interventions have been shown to be effective in improving psychological distress among adults when delivered face-to-face or in person [15-18]. Mindfulness has been defined as the “the awareness that emerges through paying attention on purpose, in the present moment, and nonjudgmentally to the unfolding of experience moment by moment” [19]. Mindfulness exercises can be performed either formally by practicing meditation regularly [16] or informally [20] by bringing awareness into all aspects of life [16]. Specifically, in Indonesia, preliminary research has found that in-person mindfulness-based interventions may enhance psychological health among Indonesian university students, including improved emotion regulation [21] and reduced insomnia [22].

A recent meta-analysis found that internet-delivered mindfulness interventions are effective in reducing psychological distress (Hedges *g*=0.22-0.44); however, most of the internet-delivered mindfulness intervention studies included in this meta-analysis were conducted in Western and high-income countries [23]. Only 6 studies involved participants from LMICs or Eastern cultural backgrounds, all of which were among Chinese populations, with encouraging evidence on the effectiveness of internet-delivered, mindfulness-based interventions for improving mental health and mindfulness [24-26]. Thus, there is a significant gap in the development, evaluation, and provision of internet-delivered mindfulness interventions for university students from other LMICs and Eastern cultural backgrounds, such as Indonesia.

Research has shown that developing culturally attuned interventions will provide new knowledge about the acceptability and utility of interventions across diverse groups [27]. Mindfulness-based interventions appear to be compatible with contemporary Indonesian culture. Many participants who practice mindfulness identify meditation practice as a spiritual process and a way of connecting with their deeper self or with something far greater than themselves [28]. Thus, for an Indonesian society that strongly emphasizes religious practice [29] and spirituality (eg, acceptance and gratitude) for well-being [29,30], practicing mindfulness may be considered relevant. The largest ethnic group in Indonesia, the Javanese, also practice *nrimo* or accepting [31]. These ideas are comparable with those of mindfulness. *Nyepi*, an Indigenous annual tradition for Indonesia’s Hindu population, includes some silent meditative practices and is also said to contribute to enhancing one’s spirituality [32].

Although mindfulness practice may help people connect with their spiritual beliefs and practices [28], it originally stemmed from Buddhist meditation practice. Thus, many studies in other LMICs examining the effectiveness of mindfulness-based interventions have been conducted in countries with some dominant practice of Buddhism, such as China; Thailand; Vietnam; or other populations that are familiar with meditation practice, such as India [33]. In Indonesia, the dominant religious practice is not Buddhism, with approximately 85% of the population holding Islam as their faith, followed by Christianity, Catholicism, and Hinduism [34].

Studies have reported favorably on the acceptability of mindfulness, especially once the therapist or client understands the concept of mindfulness from the client’s spiritual perspective [35]*.* For example, in the Islamic faith system, there is the concept of *khushu*, which means humility and presence of mind during praying or *shalat*, as well as the concept of *sabr*, which is being patient, related to being nonreactive when experiencing difficult emotions [36]. Other Islamic religious practices that are considered relevant to mindfulness practices are *muroqobah* (reflection), gratitude, seclusion or silence, *Dhikr*, and Qur’an recitation [37]. In addition to Islam, the practice of mindfulness could be relevant to other religions. For instance, in Christianity and Catholicism, there are practices of “centering prayer,” a method of meditation that places a strong emphasis on inner silence, surrender, and *lectio devina* (meditation that involves reading scripture slowly and deliberately) [38,39]. In Hinduism, *gayatri mantra* [40], yoga, and focusing the mind on *Atman* and *Paratman* are also related to mindfulness practices [37]. Thus, it is conceivable that developing culturally attuned internet-delivered mindfulness interventions may be beneficial for Indonesian society, which comprises various multicultural and multireligious backgrounds.

Cultural adaptation of existing evidence-based interventions is important for several reasons. First, cultural adaptation prevents developers from “reinventing the wheel” [41] and investing substantial time and resources developing a new intervention. Second, treatments that have already been shown to be effective can be adjusted for the targeted group, and the outcomes of the adjusted program can be benchmarked against previous findings. Third, culturally tailored interventions may increase treatment adherence [42]. Furthermore, adapting evidence-based psychotherapeutic interventions to harmonize with relevant spiritual and cultural worldviews can enhance their benefits and applicability among diverse populations [36,43,44].

Furthermore, although some Indonesian internet-delivered mental health apps are available (eg, Riliv and KALM), developing and evaluating an Indonesian, structured, module-based, and internet-delivered mindfulness intervention program is novel. Accordingly, we decided to culturally adapt an Australian, evidence-based, and internet-delivered mindfulness intervention, *Introduction to Mindfulness* from This Way Up [45]. In this paper, we report on and describe the process of cultural adaptation based on the heuristic systematic cultural adaptation framework by Barrera and Castro [46]. Describing the detailed process of cultural adaptation of internet-delivered interventions is imperative as they have usually been poorly reported in previous studies [47].

### Goals and Significance of the Study

This study sought to develop a culturally adapted internet-delivered mindfulness intervention for Indonesian university students experiencing psychological distress. In this paper, we illustrate and describe the process of culturally adapting an Australian internet-delivered mindfulness intervention, *Introduction to Mindfulness* from This Way Up [45,48], into a culturally relevant Indonesian internet-delivered mindfulness program for university student distress.

## Methods

### Intervention to Be Adapted

**Figure 1:**
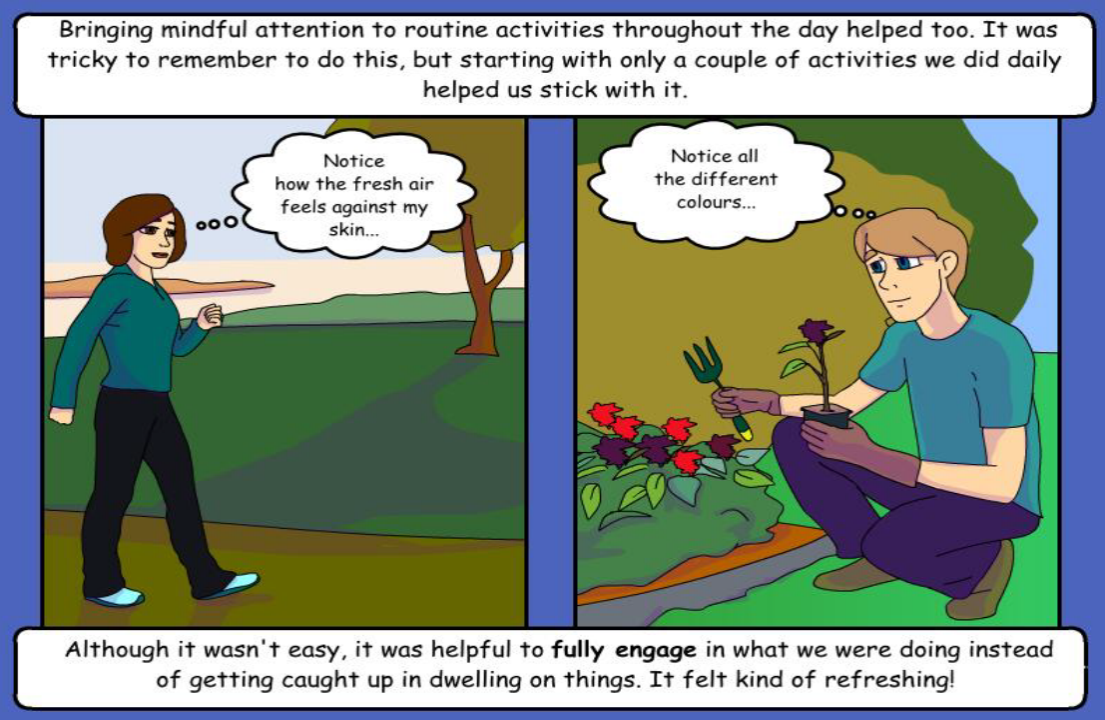
A sample illustration from the This Way Up Introduction to Mindfulness program.

The 4-lesson *Introduction to Mindfulness* program from This Way Up is a free internet-delivered self-help mindfulness program originally developed in Australia for the general English-speaking population [45]. The program describes the journey of 2 fictional characters learning mindfulness skills to improve their well-being. Each lesson comprises a set of comic-style lesson slides that introduce the concept of mindfulness and how it can help reduce psychological distress (see the example in Figure 1) as well as a summary workbook with homework activities, a daily mindfulness journal, and a list of mindful daily activities. The program also contains 7 audio-guided mindfulness meditations adapted from mindfulness-based cognitive therapy and mindfulness-based stress reduction that can be downloaded to help participants practice mindfulness [45].

### Systematic Cultural Adaptation Framework

Bernal and Domenech Rodríguez [49] explained that cultural adaptation refers to systematic changes to an intervention protocol that consider language, culture, and context (including persons, metaphors, contents, concepts, treatment goals, and methods) that are relevant to the client’s cultural patterns, meanings, and values, with the intention of increasing the congruence between the client’s cultural context and evidence-based intervention protocols [49,50].

To do so, Kreuter et al [51] suggested that researchers and clinicians can apply strategies that include packaging the health promotion or intervention in ways likely to appeal to the target group (*peripheral strategies*); highlighting the perceived relevance of a health issue (*evidential strategies*); providing the program in the dominant or native language of the target group (*linguistic strategies*); involving indigenous trained para-professionals, lay community members, or other “natural helpers” from the target group to participate in program planning and development (*constituent-solving strategies*); and embedding cultural values, beliefs, and behaviors in the program to provide context and meaning to a given health problem or behavior (*sociocultural strategies*).

To support the systematic application of the cultural adaptation strategies, we used the work by Barrera and Castro [46] as the main framework for developing cultural adaptations of behavioral health interventions. This framework consists of five stages: (1) information gathering, (2) preliminary adaptation design, (3) preliminary adaptation tests, (4) adaptation refinement, and (5) adaptation trial [52]. This paper will explain the first 2 stages of the cultural adaptation process, namely, information gathering and preliminary adaptation design. In this study, we used a mixed methods design combining quantitative and qualitative research methods [53].

### Ethics Approval

Study 1 was approved by the University of New South Wales Human Research Advisory Panel C Human Research Ethics Committee (HC3277) and the Indonesian Consortium of Psychological Science Ethics Committee (019/2020 Etik/KPIN). Study 2 received different ethics approval with study 1; it was approved by the University of New South Wales Human Research Ethics Committee (HC 3420) and the Indonesian Consortium of Psychological Science Ethics Committee (019/2020 Etik/KPIN).

### Information Gathering—Study 1: Indonesian University Students’ Preferences Regarding an Internet-Delivered Mindfulness Program

#### Overview

The aim of study 1 was to examine preferences, needs, and willingness to use an internet-delivered mindfulness program among Indonesian university students. We hypothesized that Indonesian students would rate an internet-delivered mindfulness program as acceptable and feasible to use during their university studies.

This study involved a cross-sectional, web-based survey of Indonesian university students. The survey was conducted in February 2020 using the Qualtrics platform (Qualtrics International Inc). Participants were offered the opportunity to enter a gift card draw for 1 of 10 US $16 gift cards.

#### Recruitment

The inclusion criteria were university students (1) identifying as Indonesian studying either in Indonesia or Australia, (2) fluent in writing and speaking Indonesian, and (3) having internet access. We recruited participants from the community during February 2020 via invitations through social media announcements (eg, WhatsApp, Instagram, and Facebook) from Indonesian student groups and Indonesian mental health communities (eg, the Instagram account of Seribu Tujuan and Belajarpsikologi.id), professional or psychological science associations (eg, Indonesian Association of Health Psychology and Indonesian Consortium of Psychological Science), and other internal university student WhatsApp groups across public and private universities (eg, Universitas Indonesia, Universitas Islam Indonesia, and Universitas YARSI).

#### Measures

Participants were asked to answer 2 self-report questionnaires. The first part comprised questions about their demographic information (ie, gender, age, and study level), experience using the internet in daily life, and a question to identify and choose the most important health and well-being issues for university students in their opinion from a list provided (ie, stress management, mental health issues, time management, and sleep problems). In the second part, participants answered questions about their program preferences in terms of duration, number of modules, format, content, and platform. They also chose their degree of interest in using an internet-delivered mindfulness program for improving their psychological health (1=*not interested at all*, 2=*somewhat interested*, 3=*quite interested*, and 4=*interested*). The answers were then categorized to describe the students’ openness to an internet-delivered mindfulness program. The students who chose “somewhat interested,” “quite interested,” and “interested” were categorized into “have openness to the internet-delivered mindfulness program.” Those who chose “not interested at all” were categorized as “not open to have an internet-delivered mindfulness program.”

#### Statistical Analysis

Using SPSS (version 20; IBM Corp), descriptive statistics summarized participants’ demographics and preferences regarding internet-delivered mindfulness programs.

### Preliminary Design—Study 2: Investigating Stakeholder Recommendations and Culturally Adapting an Internet-Delivered Mindfulness Intervention

#### Overview

Study 2 aimed to develop an initial adapted program, examine its preliminary acceptability among key stakeholders, and subsequently refine the program. We hypothesized that there would be several components of the internet-delivered mindfulness program that would require adjustments based on stakeholders’ recommendations. This stage was conducted between July 2020 and September 2021.

#### Preliminary Adaptation

First, the *Introduction to Mindfulness* program was translated into Indonesian by RAL, a native Indonesian senior clinical psychologist with proficient English (overall academic International English Language Testing System Band=7.5 of 9). Second, to accommodate a brief but culturally attuned program, we reduced the 2 main characters to 1 and changed the name of the main character from the English version (ie, Liz) to an Indonesian name (Lisa). Third, as the original intervention was developed for adults in general, we adjusted the main character’s story to be more relevant to Indonesian university students. Specifically, we changed life stressors from work and financial stressors to the academic and family problems typically experienced by university students. However, there was no adjustment to the actual mindfulness-based concepts and skills or the theoretical framework of the original intervention. This process was conducted between July 2020 and January 2021.

#### Recruitment of Stakeholders

Study 2 involved two groups of stakeholders: (1) university students as the end users of the mindfulness program and (2) mindfulness or mental health practitioners for providing expert feedback.

During February 2021, we recruited university students as well as mental health experts and mindfulness practitioners to participate in our study using the same recruitment strategy as in study 1.

After reading the advertisement and providing consent, potential participants completed a web-based expression of interest via Qualtrics. The expression of interest questionnaire asked for participants’ demographic information, educational background, contact details, and experience with mindfulness practice as well as requesting consent to participate in the study. In total, 71 university students and 28 mental health and mindfulness practitioners completed an expression of interest.

We then screened interested participants based on the study inclusion criteria. The inclusion criteria for the mental health and mindfulness practitioners were (1) experienced clinical psychologist, mental health counselor, or mindfulness practitioner with ≥2 years of clinical experience; (2) experience either using mindfulness-based interventions in professional practice or working with university student populations; (3) understanding of Indonesian culture and ability to speak Indonesian; (4) access to the internet using a computer, smartphone, or tablet; and (5) willingness to participate in a focus group or interview using videoconferencing software platform. For Indonesian university students, the inclusion criteria were (1) Indonesian university students with the ability to speak Indonesian fluently, (2) age of ≥18 years, (3) access to the internet and digital technology (computer, smartphone, or tablet), and (4) willingness to participate in a focus group or interview using videoconferencing software platform.

From all the participants who fulfilled the inclusion criteria, we selected a sample of students purposely to obtain feedback from students with a variety of personal, professional, and study backgrounds. For university students, we sought to ensure that the selected participants varied in terms of their age, level of study (postgraduate or undergraduate), ethnic background, religion, and study major (psychology vs nonpsychology students). For the expert participants, we tried to ensure that they had diverse characteristics related to the duration of their mindfulness practice, professional background, clinical expertise, gender, and religious background. The selected participants were then invited by email to join interviews and focus group discussions.

#### Procedures of Engagement

##### Overview

The initial translation of the first mindfulness lesson that was originally developed at the beginning of study 2 was shown to all the stakeholders. Stakeholders provided feedback and recommendations via (1) an independent review of the program and completion of the Cultural Relevance Questionnaire (CRQ; Salamanca-Sanabria et al [54]) and then (2) semistructured interviews or focus group discussions. This process of engagement with stakeholders took place between February 2021 and March 2021. All the participants in study 2 were compensated with an e-wallet top-up of A$50 (US $33.20) or IDR 500,000 (US $33.05).

##### Stakeholder Independent Review of the Program

All participants independently reviewed the draft program (which was sent via individual email). The program draft included (1) the initial translated comic-style script of the first mindfulness lesson about the main character (Lisa) deciding to practice mindfulness, (2) sample lesson 1 homework activities (eg, daily mindfulness journals and list of daily mindfulness activities), (3) scripts for audio-guided meditations in the first session (3-minute breathing space and raisin practice), and (4) a sample of the comic-style illustration from the original English version (Figure 1) to help stakeholders get a general idea of how the comic illustration style psychoeducation content is delivered. Participants were given 1 week to complete their review.

##### Completion of CRQ

Following their independent program review, participants completed the general assessment part of the CRQ [54], which contains 5 self-report questions that evaluate the relevancy of a culturally adapted psychotherapy protocol related to some areas (functional, conceptual, and linguistic). In this case, we adapted the questions to be relevant to our study. First, regarding functional relevancy, we asked whether the internet-delivered mindfulness program involved familiar behavioral or emotional expressions (such as appropriate facial expressions when feeling sad or when coping with stress activities) among Indonesian university students. Second, we asked whether treatment goals were explained using examples, personal stories, and tools consistent with Indonesian university students’ values. Third, we asked whether the social, geographical, and economic context of being an Indonesian university student was appropriately reflected in the program. Fourth, related to conceptual relevancy, we asked whether the symbols (greetings and metaphors) and treatment concepts in the program (ie, being accepting, mindful, compassionate toward oneself, and nonjudging) were also relevant to Indonesian students. Fifth, linguistically, we asked whether the oral and written language in the program could be understood and relevant in the Indonesian context**.** Respondents rated the relevancy of the items on a 5-point Likert scale (from 1=*not relevant at all* to 5=*very relevant*).

##### Focus Groups and Interviews

After completing the CRQ and independent program review, expert participants attended an individual interview (to accommodate time and scheduling limitations), and students attended a focus group discussion (3 groups of 4-8 participants each). All discussions were held between February 2021 and March 2021 and conducted over the Zoom (Zoom Video Communications) videoconferencing platform (because of COVID-19–related restrictions). The groups and interviews lasted 60 to 90 minutes and were led by RAL with the help of AA and another research assistant. Interview and focus group data were recorded digitally (using Zoom) and transcribed by AA and a research assistant.

The interviews and focus groups explored participants’ opinions on (1) the content and structure of the draft program, (2) which components needed modification, and (3) culturally sensitive issues related to mindfulness and distress. In addition, students discussed their experiences of stress, including what commonly triggers stress among Indonesian university students and how mindfulness might be relevant for students handling everyday distress. The full list of discussion questions can be obtained by contacting the first author (RAL).

#### Analysis

Descriptive statistics using SPSS (version 20; IBM Corp) summarized quantitative CRQ data. Responses from the interviews, focus groups, and CRQ qualitative data were analyzed using thematic analysis by reading and rereading the transcripts, coding responses into main themes and subthemes, and then summarizing themes and subthemes across transcripts to determine the consistent and key recommendations provided by the stakeholders as a group. Categorization was conducted by RAL as principal investigator and then consolidated through feedback and discussion with one of the Indonesian team members (CK) as second rater. The summary was a consensus between the 2.

## Results

### Study 1: Indonesian University Students’ Preferences Regarding an Internet-Delivered Mindfulness Program

#### Sample

Of the 356 respondents who initially accessed the survey link, 69.7% (248/356) completed all the survey questions (mean age 24.23, SD 6.29; range 17-56 years). More women (178/247, 71.8%) than men (70/248, 28.2%) completed the survey. Most were undergraduate students (144/248, 58.1%) compared with postgraduate or research students (124/248, 41.9%). Furthermore, most participants were studying in Indonesia (199/248, 80.2%) compared with overseas (49/248, 19.8%), and most respondents (57/248, 54.9%) reported that they typically used the internet for >6 hours each day.

#### Outcomes

##### Program Content

Respondents reported that the top 3 concerns were time management (168/248, 67.7% of respondents), stress management (148/248, 59.6%), and mental health problems (depression, anxiety, trauma, and self-harm; 144/248, 58.1%).

##### Openness to Internet-Delivered Mindfulness Program

Most respondents (240/248, 96.8%) reported openness to an internet-delivered mindfulness program. In this regard, 45.6% (113/248) indicated that they were “quite interested,” 33.5% (83/248) were “interested,” and 17.7% (44/248) were “somewhat interested” in doing an internet-delivered mindfulness program. Only a small number of participants (8/248, 3.2%) stated that they had no interest at all.

##### Delivery Format

Among the participants who were interested in an internet-delivered, mindfulness-based program (240/248, 96.8%), most (201/240, 83.8%) preferred a program that involved guidance and feedback (vs self-help).

##### Duration, Number of Modules, and Platform

Among the participants who were interested (240/248, 96.8%), most (127/240, 52.9%) preferred the length of the program to be 3 to 4 sessions, with most (110/240, 45.8%) preferring that each lesson take only 15 to 30 minutes to complete. Most interested participants (194/240, 80.8%) wanted the program to be accessible through either a website or mobile phone app compared with preferring a mobile app only (36/240, 15%) or website access only (10/240, 4.1%).

These preliminary results indicate that Indonesian university students are open to the idea of completing an internet-delivered mindfulness intervention that is tailored to address their daily concerns. Students could identify their preferences for the duration, format, and delivery of the intervention, and these were considered during the cultural adaptation of the program.

### Study 2: Stakeholder Recommendation and Cultural Adaptation of an Internet-Delivered Mindfulness Intervention

#### Sample

The sample of expert participants comprised 4 clinical psychologists and 2 mindfulness teachers or practitioners (mean age 39.00, SD 8.62; range 28-47 years). The experts included 33% (2/6) men and 67% (4/6) women, and they represented various religions (2/6, 33% Muslim; 1/6, 17% Christian; 1/6, 17% Catholic; 1/6, 17% Hindu; and 1/6, 17% Buddhist) and ethnicities (3/6, 50% Javanese; 1/6, 17% Chinese; and 2/6, 33% Balinese). Expert participants reported practicing mindfulness personally and teaching it professionally for 3 to 17 years.

Regarding the university student sample, 20 university students were invited to join the focus group discussion. In total, 95% (19/20) of the university students (mean age 23.79, SD 4.7 years; 9/19, 47% women and 10/19, 53% men) responded to the invitation and then completed their independent review of the draft program and CRQ. However, 11% (2/19) of the students were unable to attend a follow-up focus group, and consequently, the final student sample comprised 17 students (7/17, 41% women and 10/17, 59% men; mean age 24.12, SD 4.6; range 18-35 years). They reported a variety of religious backgrounds (14/17, 82% Islamic; others were Catholic [1/17, 6%], Christian [1/17, 6%], and Hindu [1/17, 6%]) and ethnicities (5/17, 29% Javanese and 3/17, 18% Sundanese, with the remaining 9/17, 53% individually identifying as Minangnese, Bataknese, Chinese, Muna, Acehnese, and Palembangnese). Participants were also completing a range of educational majors, including psychology (4/17, 24%), engineering (5/17, 29%), social sciences such as communication or political science (2/17, 12%), linguistics and education (2/17, 12%), nursing and pharmacy sciences (3/17, 18%), and biological sciences (1/17, 6%). Most participants (10/17, 59%) reported familiarity with mindfulness.

During the focus groups, participants were divided into 3 groups based on their time availability to join the discussion. Group 1 comprised 47% (8/17) of the students, group 2 comprised 29% (5/17) of the students, and group 3 comprised 24% (4/17) of the students. Details of participants’ characteristics in each group are shown in Table 1.

**Table 1:** University students’ characteristics during the focus group discussion (n=17).

Category			All		Group 1 (n=8)		Group 2 (n=5)		Group 3 (n=4)
Age (years), mean (SD)			24.12 (4.6)		23.25 (4.4)		25. 8 (6.38)		23.75 (2.7)
**Gender, n (%)**									
	Men	10 (59)		5 (62)		2 (40)		3 (75)	
	Women	7 (41)		3 (38)		3 (60)		1 (25)	
**Educational level, n (%)**									
	Undergraduate (bachelor’s or vocational degree)	9 (53)		5 (62)		2 (40)		2 (50)	
	Postgraduate (master’s or doctoral degree)	8 (47)		3 (38)		3 (60)		2 (50)	
**Major of study, n (%)**									
	Psychology	4 (24)		1 (12)		2 (40)		1 (25)	
	Engineering	5 (29)		2 (25)		1 (20)		2 (50)	
	Health sciences (ie, nursing and pharmacy)	3 (18)		2 (25)		1 (20)		0 (0)	
	Social sciences (ie, communication and political sciences)	2 (12)		1 (12)		0 (0)		1 (25)	
	Linguistics and education	2 (12)		2 (25)		0 (0)		0 (0)	
	Others (ie, biological science)	1 (6)		0 (0)		1 (20)		0 (0)	
**Religious background, n (%)**									
	Islamic	14 (82)		7 (88)		5 (100)		2 (50)	
	Catholic	1 (6)		1 (12)		0 (0)		0 (0)	
	Christian	1 (6)		0 (0)		0 (0)		1 (25)	
	Hindu	1 (6)		0 (0)		0 (0)		1 (25)	
**Ethnicity, n (%)**									
	Javanese	5 (29)		3 (38)		1 (20)		1 (25)	
	Sundanese	3 (18)		1 (12)		1 (20)		1 (25)	
	Other (eg, Minangnese, Bataknese, Chinese, Muna, Acehnese, and Palembangnese)	9 (53)		4 (50)		3 (60)		2 (50)	
**Previous knowledge of mindfulness, n (%)**									
	Yes	10 (59)		5 (62)		3 (60)		2 (50)	
	No	7 (41)		3 (38)		2 (40)		2 (50)	

#### Quantitative Evaluation on the Basis of CRQ

Expert participants rated (from 1=*not relevant at all* to 5=*very relevant*) the relevancy of the initial translated version of the internet-delivered mindfulness lesson to contemporary Indonesian culture using the CRQ.

On average, they reported that the initial draft program was relevant to Indonesian culture with respect to behavioral and emotional expression (mean 4.67, SD 0.51), treatment goals (mean 4.33, SD 1.03), and context (mean 4.33, SD 1.03). Experts also considered the program to be conceptually (mean 4.5, SD 0.83) and linguistically (mean 5.00, SD 0.0) relevant to Indonesian populations.

The 19 university students who completed the CRQ also perceived the initial internet-delivered Indonesian mindfulness module to be highly relevant to Indonesian culture with respect to its behavioral and emotional expressions (mean 4.53, SD 0.61), treatment goals (mean 4.53, SD 0.69), context (mean 4.26, SD 0.65), concept (mean 4.63, SD 0.49), and language (mean 5.00, SD 0.00).

These adequate cultural relevancy scores (all >4) suggest that experts and university students perceived that the adapted program contained behavioral or emotional expressions that were relevant to Indonesian culture; the treatment goals reflected Indonesian values; and the context was consistent with the social, geographical, and economic situation among Indonesian populations. The program was also perceived as having symbols (greetings and metaphors) and treatment concepts that were relevant to Indonesian students. Finally, linguistically, the program’s language was understandable and appropriate in an Indonesian context.

#### Qualitative Recommendations and Key Decisions Related to Cultural Adaptation

##### Overview

Qualitative data from the CRQ, interviews, and focus groups were thematically analyzed. We categorized stakeholder recommendations into two domains that consisted of feedback regarding (1) the cultural relevancy of the program and (2) the practical or delivery aspects of the intervention. The following sections present the highlights of the analysis and key decisions made to modify the program.

##### Cultural Adaptation of the Intervention Content

Following Bernal et al [55], we categorized participants’ feedback according to categories such as the program’s language, metaphors, or symbols, persons, content, concepts, contexts, methods, and goals (see Multimedia Appendix 1).

##### The Practical and Delivery Aspects of the Program

Stakeholders’ feedback related to the program’s practical and delivery aspects and the decisions made when developing the new Indonesian program are summarized in Multimedia Appendix 2.

To summarize, based on stakeholders’ input from the web-based survey, interviews, focus group discussions, and CRQ responses for its preliminary design, we modified the original *Introduction to Mindfulness* to create an Indonesian internet-delivered mindfulness program called *Program Intervensi Mindfulness Daring Mahasiswa Indonesia* (*PSIDAMAI*)*.* During the process of cultural modification and translation for the full program, RAL, as an Indonesian senior clinical psychologist, was assisted by AA, an Indonesian registered psychologist with proficient English ability (Test of English as a Foreign Language score of >550).

Final version of *PSIDAMAI* comprises 4 self-paced internet-delivered modules that teach university students to practice mindfulness over 4 weeks of intervention. Although the main treatment content, internal logic processes, and other essential components of the mindfulness-based intervention were all retained from original intervention, necessary cultural modifications and practical enhancements (ie, illustration and delivery format) were made to suit the Indonesian context. It includes the journey of a fictional character using a comic-style illustration. These illustrations featured Indonesian characters and narratives. Additionally, the incorporation of mindful activities into daily life encompassed a broader range of social and spiritual activities. Modifications were made to psychoeducational lessons to reduce their duration, and these lessons were delivered using written text, visual illustrations, along with audio tracks. The design is different from to the original version, which only utilized visual illustrations. To increase adherence and engagement, we also plan to give guidance for the participants using internet-delivered platforms that they preferred (either by SMS, emails, or WhatsApp). Participants could receive phone consultation based on their request. We will also give certificate and reward for participants who completed the program (see details in Multimedia Appendix 2).

**Figure 2:**
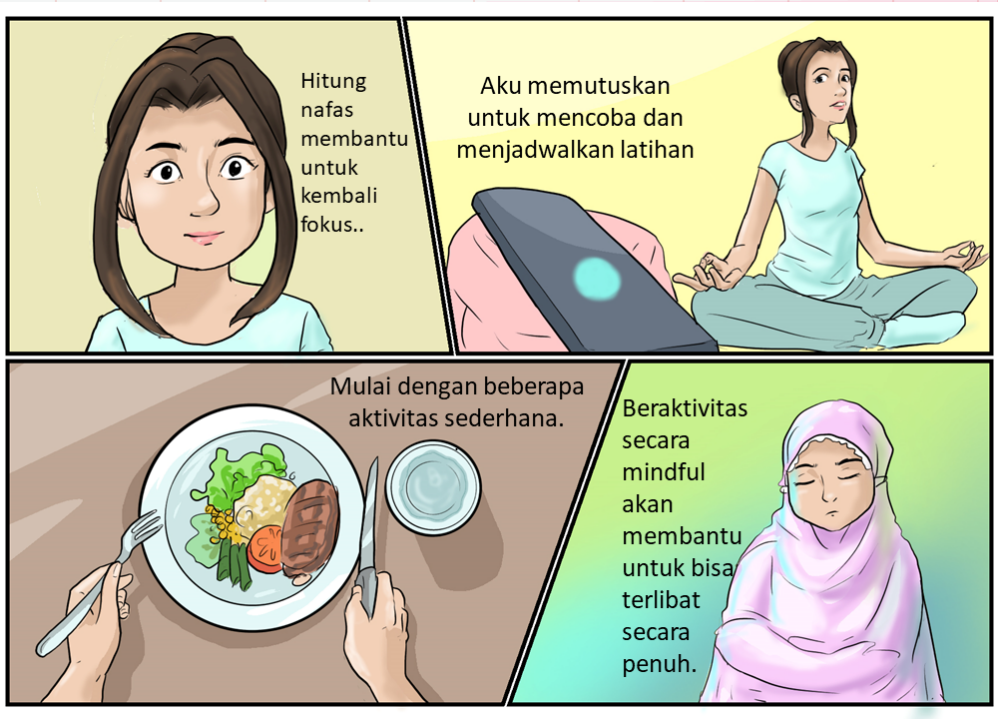
Sample illustration from Program Intervensi Mindfulness Daring Mahasiswa Indonesia—an Indonesian Internet-delivered mindfulness intervention for university students.

The entire cultural adaptation process at this stage lasted approximately 5 months, from April 2021 to September 2021. A sample of the final adapted program can be accessed by contacting the first author. Figure 2 provides a sample illustration from *PSIDAMAI*.

## Discussion

### Principal Findings

Scalable, evidence-based, and culturally appropriate psychological interventions are essential to address the mental health needs of people worldwide. By reporting the process of cultural adaptation transparently, we gain insights into which components of interventions are culturally relevant and which require adjustment [56]. The details of cultural adaptation of internet-delivered programs have typically been underreported [47], and this study highlights the importance of creating programs, particularly mindfulness programs, that are relevant to a targeted population.

In this paper, we detailed the implementation of the first 2 stages of the framework by Barrera et al [46,52]—information gathering and preliminary design—as applied to a brief internet-delivered mindfulness program such that future researchers can replicate and refine these methods. Integrating the cultural elements (ie, language, symbols, metaphors, content, treatment goals, person, methods, context) proposed by Bernal et al [55], we adapted the Australian *Introduction to Mindfulness* program for adults and created *PSIDAMAI*, a culturally attuned program for Indonesian university students.

This study also highlights the utility of the strategies proposed by Kreuter et al [51] for enhancing the cultural appropriateness of health interventions. During the adaptation, we applied peripheral strategies that involved packaging the program based on the preferences of end users (eg, creating a brief program with an attractive name and including visual and audio-based information). We also applied evidential strategies; by presenting evidence of the program’s potential impact, we sought to enhance the perceived relevance of mindfulness interventions to the stressors and challenges faced by university students. Linguistic strategies were also applied by providing the program in everyday or common Indonesian language.

We relied heavily on constituent-involving strategies that involved seeking comprehensive program recommendations from local Indonesian mental health experts and university students from different religious and ethnic backgrounds. As predicted, multiple aspects of the Australian program required modification, and Indonesian stakeholders were able to provide clear guidance on how to enhance the cultural relevancy of the treatment.

Most of Indonesian society consists of different characteristics (various religious and ethnic backgrounds) compared with where mindfulness-based intervention is derived from (which is Buddhist meditation). Thus, including Indonesian experts and students from different backgrounds in the cultural adaptation process is very important.

Our study suggested that mindfulness-based interventions are generally acceptable among people from different backgrounds in Indonesia with some adjustments to the delivery and introduction of the concept of mindfulness practice. In this case, we applied sociocultural strategies by embedding the cultural values, beliefs, and behaviors of typical Indonesian populations to provide an appropriate context and meaning for mindfulness practices and their use among distressed Indonesian university students. On the basis of the preference survey results, and considering the collective values among Indonesian populations, we also plan to use Indonesian lay counselors to guide and support students during the intervention.

In study 1, we found that the top 3 mental health and well-being issues reported by our student sample were time management, stress management, and depression or anxiety. This finding is consistent with previous research in Western and high-income countries such as Australia that found that work-life balance (time management), stress, and mental health issues (depression and anxiety) are priority concerns among university students [57]. Our results also suggest that most Indonesian university students are open to trialing an internet-delivered mindfulness program. Similar findings have been previously reported in a study that showed that Indonesian populations were generally open to internet-based interventions for treating mental health problems [58]. Compatible with a previous cultural adaptation study of a web-based stress management program for Indonesian university students [41], we also found that users preferred short, mobile phone–friendly modules. Participants in study 1 also preferred to have a guided program compared with self-help programs. This is relevant as a previous meta-analysis of internet-based programs found that guided programs may be more effective compared with self-help programs [59].

In study 2, we found evidence that our culturally adapted internet-delivered mindfulness intervention was considered relevant for Indonesian university students with some cultural and delivery adjustments. This was reflected in participants’ CRQ responses, which indicated that our initial draft program was perceived as highly culturally relevant (mean rating range 4.26-5.00, where 5=*very relevant*). Nevertheless, participants’ qualitative feedback indicated that aspects of the program required adjustment.

We found that recommendations from stakeholders were consistent with findings from previous cultural adaptation studies of internet-delivered interventions in other low-resource settings, including Indonesia. For instance, preferences for simplified language and the use of audiovisual content rather than text are similar to earlier studies among university students in Indonesia and Colombia [41,60]. Some suggestions to include spiritual, social, and religious activities in the new mindfulness program were also similar to the findings of studies adapting internet-delivered behavioral activation [61] and transdiagnostic internet-delivered cognitive behavioral therapy for Indonesian populations [62]. However, adaptations related to the concept and practice of mindfulness were unique to this study. For instance, given our understanding of the religious and spiritual values and semantics of Indonesian society, we chose to retain the term “mindfulness” (instead of using the Indonesian term *rasa berkesadaran*) and avoided the term “meditation” (instead preferring the general term “mindfulness practice” or *latihan mindfulness* in Indonesian). Although the evidence-based theoretical and clinical aspects of the Australian mindfulness program were not altered, this study demonstrates how adjusting the style, delivery, and language of health interventions can enhance their relevance and sensitivity among culturally diverse people.

### Implications

In addition to highlighting the importance of adapting an evidence-based internet-delivered intervention to be more relevant to the targeted population, there are multiple implications to consider from this study, especially with respect to the challenges that researchers and clinicians may encounter during the cultural adaptation process.

First, even though various mental health apps are available in Indonesia, the idea of structured internet-based mindfulness interventions was unfamiliar to our participants from Indonesia. Therefore, during the process, it is necessary to describe the program in depth to ensure that participants grasp the structure and concepts of internet-delivered mindfulness interventions to provide appropriate feedback and suggestions for modification.

Second, many people in Indonesia lack a dependable and quick internet connection. Thus, the inclusion of audio and visual components in the intervention platform should be carefully considered. Our decision to choose static illustrations with brief text and optional audio lessons (instead of streaming videos) accommodates local internet limitations while also offering choice and supporting the learning preferences of end users who favor visual and image-based content. Similarly, the platform on which internet-based programs are provided needs to be accessible to end users. In this case, students desired a mobile-friendly platform that worked well within a university context.

A third issue that needs to be highlighted is the importance of choosing a program platform that works well within the consumer’s context, in this case, in the university context. Ideally, all the program modules, including support that is provided to participants during the internet-delivered intervention, are designed inside the platform itself. However, to accommodate participants’ needs related to bidirectional support and feedback using a mobile phone–friendly platform, we decided to incorporate guidance through direct communication by other internet-delivered platforms based on participant preferences (either by email, WhatsApp, or SMS text messages). Participants could also request a non–face-to-face phone consultation if they had difficulties with the program or mindfulness practice.

Finally, although participants reported preferring a clinician-guided program involving bidirectional support and feedback, the availability of mental health professionals in Indonesia is limited. Given the university context for our work, guidance during program completion would be provided by appropriately supervised psychology or counseling students or graduates instead of by fully licensed mental health professionals. The scope of the tasks performed by counselors can be limited to prompting program completion and encouraging regular mindfulness practice. Support can also use communication methods that are accessible and familiar to and preferred by Indonesian students (eg, email, WhatsApp, SMS text messages, or phone calls). When needed, students could be referred to licensed psychologists to have more clinical support. This stepped-care monitoring support was also found to be beneficial in a previous study on an internet-delivered behavioral activation for depression in Indonesia [12].

### Limitations and Future Directions

This study has some limitations. First, the number of students and experts providing detailed feedback and program recommendations was not large. Although we selected participants with diverse demographics, it is unclear how representative our participants’ feedback is of that of the broader Indonesian population. Our results regarding students’ main concerns might not represent university students in general, especially those who have more limited internet access and come from remote regions or lower socioeconomic backgrounds. We did not capture the financial and economic background of the participants. As financial issues could be potential stressors for individuals coming from lower economic backgrounds, future studies could assess participants’ socioeconomic background and its association with their attitude toward internet-delivered programs, as well as their main concern as students.

Second, in our study, we did not collect data on the level of psychological distress among student participants or specifically target participants who had high levels of distress. It is possible that students experiencing high distress may require specific program adaptations as previous studies have found that the needs and concerns addressed by internet-delivered intervention programs are related to the level of distress experienced by program users [57]. Thus, in future cultural adaptation studies among Indonesian students, researchers could include measurement of participants’ distress to examine whether different distress levels are associated with different feedback responses related to the adaptation of programs.

Third, even though we tried our best to follow the systematic guidelines for adapting the intervention, we realized that our adaptation process had some limitations. There are some ideal principles and processes of cultural adaptation that were not feasible because of time and financial constraints. For instance, during the translation process, we only involved an English-proficient Indonesian psychologist and not a panel of translators from different backgrounds. We engaged with our stakeholders subsequent to the translation process to ensure the linguistic relevancy of the program. When feasible, future studies are recommended to include a panel of translators as well as complete a back translation process to help minimize bias [63]. It would also be ideal if the illustration adaptation was started before the qualitative study began. To further ensure cultural attunement, participants would also have to review the entire draft mindfulness program (not just the first lesson) before providing feedback and then would have to provide a second round of feedback after reviewing the revised program.

The influence of the global context and timing of data collection could be considered in future studies. Our study was carried out during the COVID-19 lockdown period (between 2020 and 2021), which may have influenced our findings. First, we needed to use a videoconferencing platform for the data-gathering method in our qualitative study, which limited our interaction with and observation of participants during the interviews and discussions. Second, the perspective of the participants related to the internet-delivered intervention and psychological distress might be different than if it had been conducted after the pandemic period.

In study 1, we found that time management was a major concern for university students alongside stress and mental health issues (depression and anxiety). Although our internet-delivered program highlighted difficulties in managing time as one of the stressors faced by students, the focus of the program was on managing psychological distress. This focus has the broadest applicability, and previous studies have found that managing stress is the greatest concern for university students with higher levels of psychological distress [57]. It is also possible that mindfulness skills can be used by students to help them better manage their time and prioritize their goals [64]. Nevertheless, future studies targeting specific concerns such as time management or financial burden might be worthwhile and relevant for program development among Indonesian university students. In addition, the information gathered in study 2 about stressors experienced by students during the COVID-19 pandemic could also be useful for the development of programs in future pandemics.

Apart from these limitations, this study provides a useful first step toward culturally adapting internet-delivered mindfulness interventions for Indonesian university students. On the basis of the theoretical framework by Barrera and Castro [46], which includes (1) information gathering, (2) preliminary design, (3) preliminary adaptation test, (4) adaptation refinement, and (5) adaptation trial, the next stage is to conduct preliminary adaptation testing, further refinement, and a subsequent clinical trial. Therefore, our next studies will examine the feasibility, acceptability, and effectiveness of *PSIDAMAI* among Indonesian university students and continue to explore its cultural relevance and attunement. The protocol for a pilot trial and randomized controlled trial of *PSIDAMAI* have been developed and can be accessed in the Australian New Zealand Clinical Trials Registry (ACTRN12622000729729 and ACTRN12621001411831), and a brief preliminary report of our pilot trial has been presented [65].

### Conclusions

Cultures worldwide are diverse and dynamic. Thus, there is an urgent need to develop and refine successful methods of cultural adaptation so that evidence-based treatments can be accessible, appropriate, and effective in multiple populations. This paper describes the initial process of culturally adapting an Australian internet-delivered mindfulness intervention into an Indonesian internet-delivered mindfulness intervention called *PSIDAMAI*. Our findings suggest that an Indonesian culturally adapted, internet-delivered mindfulness intervention for treating distress is relevant to an Indonesian university student context. However, it was necessary to adjust the delivery strategy, cultural factors, program viability, and design features. Evaluation of *PSIDAMAI*’s efficacy is now needed.

## Appendix

Multimedia Appendix 1 Summary of stakeholders’ recommendations and cultural adaptation of the intervention content.

Multimedia Appendix 2 Summary of recommendations and adaptations related to practical and delivery aspects of the intervention.

## Abbreviations

CRQ: Cultural Relevance Questionnaire
LMICs: low- and middle-income countries
PSIDAMAI: Program Intervensi Mindfulness Daring Mahasiswa Indonesia

## Data Availability

The data sets generated from and analyzed during this study are available from the corresponding author upon reasonable request.
