# Supplementary material for: Culturally Adapting an Internet-Delivered Mindfulness Intervention for Indonesian University Students Experiencing Psychological Distress: Mixed Methods Study
Source: JMIR Form Res. 2023 Aug 31;7:e47126. doi: 10.2196/47126 (PMC10502595; doi:10.2196/47126)
Supplement: Multimedia Appendix 1 [file formative_v7i1e47126_app1.docx]

| **Theme and Original Version** | **Stakeholders Recommendation** | **Cultural Adaptation** |
| --- | --- | --- |
| **Language, symbols, and metaphors** | | |
| Long written text in comic slides with some technical terms, such as “auto pilot mode” | - Stakeholders suggested using more concise, simple, popular, and understandable languages for common people. - Stakeholders stated preference for the use of the term “mindfulness” rather than translating it fully into Indonesian as there is no corresponding word in the Indonesian language that can express the whole meaning of mindfulness. - Stakeholders preferred English term since it is more popular and attractive for Indonesian young people. This was supported by student participants: “Among young people with high education, Western language is more familiar. But we can give simple representative Indonesian language on beginning*.*” - Stakeholders recommended to find alternate relevant terms for mindfulness: “There is no Indonesian language that is suitable to explain about the concept of mindfulness clearly. There’s only specific language in Javanese language (‘eling lan waspodo’), or Islamic/Arabic term (‘khushu’). But if we use this term, it might reduce the broadness and universal target for the training. Term can be used ‘rasa berkesadaran’ for introduce in the beginning, but then continuing with just mindfulness is better*.*” | - We reduced word count on lesson slides and use of everyday Indonesian language and metaphors (such as “di ujung tanduk” to translate “in the edge”) to describe distress and the perception of danger in the psychoeducation components of the program. - We retained the term “mindfulness” in the whole program but used the Indonesian-relevant term when first introducing mindfulness (eg, rasa berkesadaran). |
| Using “meditation” as the main concept to explain how mindfulness practice should be done | - Stakeholders stated that using term “meditation” should be done very carefully, because many Indonesians think “meditation” is highly related to religious practice from a specific group. In this regard, most Indonesians are Muslim and may be reluctant to join the program as “meditation” is associated with Buddhism or Hinduism. | - Instead of using “meditation,” we used the term “mindfulness exercise” to explain types of mindfulness practice. |
| Audio scripts for guided meditations contain long scripts and have many “inquiry” statements | - Stakeholders’ suggestions related to audio guide: - Reminder to use intonation and pace for the long audio script during recording - Language should be simple and easy to understand for Indonesian students to help them sustain attention - Use natural background sounds (water and birds singing) to promote relaxation - Avoid inquiries/questions and give specific directions instead - Instruction for finding suitable and comfortable postures should be clearer - Including both female and male voice options is preferable | - We revised and updated the audio guides to be more concise, use simpler language, and give specific directions (rather than inquiries). - We updated the initial audio guide instructions to encourage participants to find a comfortable place and posture to practice mindfulness. |
| **Contents** | | |
| Storylines contain general stressors relevant to adults (eg, financial and work-related stressors). | - Stakeholders stated that social and family pressures are specifically relevant to students as the main stressors (ie, expectations from families, loneliness, no support system, need of social belonging, peer pressure, critical parents, relationship breakups, adjustment to new situations, inferiority, and competitiveness at university, and comparing oneself with others’ achievement in social media/social comparison). - They suggested that stressors could be related to the COVID-19 pandemic (ie, difficulties with online learning, communication with lecturers and peers, and family study commitments). | - We updated the storyline to include more emphasis on social and family pressures as the main stressors. However, we decided not to include only pandemic-related stressors so the program would remain relevant after the pandemic. |
| No self-reflection component in the diary for recording daily mindfulness practice | - Stakeholders said that reflection is an important part of Indonesian spiritual practices. Then, in home tasks, participants should be encouraged to reflect each day on their mindfulness practice. | - We added a column for personal reflections in the daily mindfulness diary. |
| There is no specific instruction regarding the “maximum dose” of daily mindfulness practice. | - Stakeholders considered that university students will be burdened with too many mindfulness practice tasks. Thus, it suggested that one daily mindfulness activity and one formal meditation practice a day is appropriate. | - We updated the instructions for home practice to encourage short but regular mindfulness practice. |
| **Conceptual framework and treatment goals** | | |
| Mindfulness meditation does not need to be part of religious practice. | - According to stakeholders, mindfulness practices can be used within a religious and spiritual context (eg, during prayer via doing self-reflection, remembering human existence in the world, and finding meaning within situations). Thus, this relevancy should be brought into the program. | - We adjusted the storyline to include more interaction between characters discussing how mindfulness can be helpful for handling distress. - We also emphasized that many types of religious activities among Indonesians (such as praying or *shalat*) could be part of daily mindful activities. |
| There is no information that explains the difference between naming an emotion and judging an emotion. | - Stakeholders considered that people who practice mindfulness are sometimes confused regarding whether giving a name to an emotion is the same as judging that emotion, because among many Indonesians, giving a label to an emotion might be the same as judging the emotion. | - We decided that this issue will be raised when training the counselors who will guide students through the program. Answers to these questions will be provided. In the audio guides, we highlighted that it is okay to name an emotion (e.g., sadness or anger) but to do so gently, without self-judgment that it is bad or good. |
| The program emphasizes the attention and awareness aspects of mindfulness. | - Stakeholders suggested that the self-compassion/self-kindness aspects of mindfulness should be emphasized more, especially for Indonesians who are self-critical. Besides, the attention and awareness aspects in mindfulness practice could be broadened to social contexts. | - We included the importance of self-kindness during mindfulness practice and highlighted how attention and awareness skills could be applied during social interactions and in relationships. |
| The differences between mindfulness, relaxation, and hypnotherapy are not explained. | - In stakeholders’ opinion, many Indonesians still cannot differentiate mindfulness practice from relaxation techniques or hypnotherapy. | - We clarified the definition of mindfulness in the program introduction and trained counselors on how to answer this question. |
| **Person and methods** |  |  |
| The Australian illustrations depict characters that reflect Australian multicultural society. | - To make the program briefer, stakeholders suggested to use a female character as more women attend counseling services in Indonesia. Besides, using images that represent Indonesians (e.g., >80% of Indonesians are Muslim, so include a female figure wearing a hijab) would be appropriate and more suitable. | - We deiced to created 1 main female character in the story. But we also created a male character depicting a mindfulness professional to represent various genders that can practice mindfulness. - We used an Indonesian illustrator to create culturally attuned images, including a Muslim female character. |
| Participants are encouraged to practice mindfulness individually. | - It is suggested that consistency, commitment, and initiative while practicing mindfulness need to be maintained by giving participants reminders; support; and, if possible, opportunities for sharing with their peers. - Stakeholders suggested to provide some guidance contains dialogues and reciprocal communication between clinicians and clients. | - To increase engagement, we decided that counselors will provide weekly support and encouragement via SMS, text messages, or WhatssApp, during the program. - Participants can also request phone consultations. |
| The fictional character in the original version starts using meditation after observing her friend and contacting health professionals. | - Considering that mental health literacy and help seeking are low among Indonesian students, stakeholders recommended that the storyline include different ways in which the character can find mental health help (from friends to finding the information on YouTube, websites, or social media) before they directly contact mental health professionals. | - We updated the story to include how the main character finds information on mindfulness. - We also included a supporting character who recommends mindfulness. - In the storyline, the main character will also find information via social media before contacting the university psychology clinic (as there is no need for a GP^a^ referral in Indonesia). |
| **Context (socioeconomic, geographic, financial, and lifestyles)** | | |
| - Lists of mindful daily activities are provided (e.g., eating, walking in the park, and using sunscreen). - Examples that explain the process of and difficulties in practicing mindfulness use adult contexts. | - Stakeholders suggested that the list of activities needs to be more culturally relevant. In this regards, social and spiritual activities such as praying, using social media, and hanging out with friends can be added. - There are some activities, such as walking in the park, enjoying fresh air, and using sunscreen, that need to be changed as they do not fit a typical Indonesian student’s geographical, financial, and cultural context. - Stakeholders suggested to use practical, student-based examples to explain mindfulness concepts and difficulties when practicing mindfulness. | - We updated the list of mindful daily activities to be more culturally relevant. - We also added activities related to social and spiritual practices, such as praying, using social media, giving Salam (Indonesian greeting style), and talking with family or parents, as well as some hobbies related to young people, such as listening to music. - We deleted activities that were unsuitable for most Indonesian students. - We updated the storyline to emphasize specific examples of how students think and feel when practicing mindfulness. |

^a^GP: general practitioner.
