# Supplementary material for: Culturally Adapting an Internet-Delivered Mindfulness Intervention for Indonesian University Students Experiencing Psychological Distress: Mixed Methods Study
Source: JMIR Form Res. 2023 Aug 31;7:e47126. doi: 10.2196/47126 (PMC10502595; doi:10.2196/47126)
Supplement: Multimedia Appendix 2 [file formative_v7i1e47126_app2.docx]

| **Theme and Original Version** | **Stakeholders Recommendation** | **Cultural Adaptation** |
| --- | --- | --- |
| **Safety** | | |
| Automatic emails with information about help and support are sent when participants self-report high distress on program questionnaires. | - Owing to its novelty, stakeholders suggested that the program needs to be clinician guided and provide contact information for safety and support when students are highly distressed. | - We decided to develop a guided program with stepped-care monitoring. First, help will be provided by trainee psychologists or counselors, with further emergency support available from the psychologist team in Indonesia. Complex difficulties will be escalated to the principal study psychologist (RAL). |
| **Psychoeducation delivery format** | | |
| Program is delivered via comic slides with embedded text to explain the concept of mindfulness. | - Feedback related to delivery: - Combined audio and visual format would be better. - Slides should be simple but attractive. - If using video, provide subtitles to help people remain attentive. In addition, consider the internet connection that might hinder video streaming. - Avoid long written text as literacy skills and reading interest are variable among Indonesian students. - Provide format variety so students can choose to listen to the audio only or see the visual image or video. - Consider delivering content via podcast or talk show format. | - We tried to accommodate both visual and audio learning modalities in the online lesson by delivering the lessons using 3 elements: - Attractive illustrations using comic-based style with more concise text - Longer script that is written below the illustration for providing additional information - Provided audio format lesson based on the longer script. Here, we hired professional voice talent to record these audio lessons.   With this kind of format, we expected that participants can choose to listen to the audio lessons only, read the longer written text, or just see the illustration. |
| Delivery on web-based platform. | - Stakeholders suggested to create a mobile phone–friendly version, as most Indonesian students access the internet using a mobile phone compared with desktop. | - We planned to upload the program onto REDCap^a^ (a secured mobile phone–friendly survey platform). |
| Original program is accessible anytime via This Way Up. | - Stakeholders gave suggestion to recruit participants at an appropriate time for students (ie, not during university examinations). | - We coordinated with the Indonesian team and will conduct the trial to fit within students’ academic schedules. |
| **Adherence and engagement** | | |
| Participants are given a certificate for completing the lessons. | - Stakeholders suggested to give additional attractive rewards for participants who have completed the program (ie, souvenirs, vouchers, and a certificate). | - We decided to provide completion certificates and compensation to reimburse internet-based costs. |
| Program called Introduction to Mindfulness | - Stakeholders suggested to create a more relevant and attractive program name using Indonesian words. | - We decided to name the adapted intervention PSIDAMAI, which comes from the term “Psiko” (Indonesian term for “psyche” or “soul”) and “Damai” (Indonesian word for “peaceful”). PSIDAMAI is also the abbreviated form of “Program Intervensi Mindfulness Daring Mahasiswa Indonesia” (in English: Internet-delivered Mindfulness Program for Indonesian University Students). |

^a^REDCap: Research Electronic Data Capture (Vanderbilt University).
